# Effect of placebo versus prophylactic postoperative amoxicillin on post-(adeno) tonsillectomy morbidity in Tanzanian children: a two-centre, double-blind randomized controlled non-inferiority trial

**DOI:** 10.11604/pamj.2022.42.142.35540

**Published:** 2022-06-22

**Authors:** Denis Katundu, Desderius Chussi, Balthazar Nyombi, Rune Philemon, Hadija Semvua, Gerjon Hannink, Niels van Heerbeek

**Affiliations:** 1 Department of Otolaryngology, Kilimanjaro Christian Medical Centre, 3010 Moshi, Tanzania,; 2 Department of Otolaryngology, Kilimanjaro Christian Medical University College, Kilimanjaro, Tanzania,; 3 Department of Medical Laboratory Sciences, Kilimanjaro Christian Medical University College, Moshi, Tanzania,; 4 Department of Paediatrics and Child Health, Kilimanjaro Christian Medical Centre, Moshi, Tanzania,; 5 Department of Pharmacy, Kilimanjaro Christian Medical Centre, Moshi, Tanzania,; 6 Department of Operating Rooms, Radboud Institute for Health Sciences, Radboud University Medical Center, Nijmegen, Netherlands,; 7 Department of Otorhinolaryngology, Radboud Institute for Health Sciences, Radboud University Medical Center, Nijmegen, Netherlands

**Keywords:** Pediatric, otolaryngology, antimicrobial resistance, placebo

## Abstract

**Introduction:**

to date in Africa, there is limited evidence regarding the role of prophylactic antibiotics to prevent post (adeno) tonsillectomy ((A)TE) morbidity in children. As (A)TE is the most performed surgery in the pediatric population, the use of prophylactic antibiotics is likely a major factor in the development of AMR. In Tanzania, as in many other settings with limited resources antibiotics are misused and overprescribed. Potential reasons include limited stewardship and widespread use of postsurgical prophylactic antibiotics. Misuse of antibiotics might contribute significantly to the development of antimicrobial resistance (AMR).

**Methods:**

a two-centre, double-blinded randomized controlled non-inferiority trial. Subjects included children from 2-14 years of age with recurrent chronic tonsillitis and/or obstructive sleep apnoea due to adenotonsillar hypertrophy who were electively scheduled for (A)TE in two tertiary hospitals. Participants were randomly allocated to receive either placebo or amoxicillin for five days postoperatively. Primary outcome was non-inferiority of placebo compared with amoxicillin for postoperative haemorrhage (margin 5%; at 14 days) postoperative fever (margin 5%; at 7 days), and pain (margin 1 point; at 7 days). Secondary outcomes included the times required for resumption of normal diet and normal activities, and microbial recolonization of the tonsillar beds. Data were analysed according to intention-to-treat principle. Follow-up was 14 days.

**Results:**

between March 13, 2019 and September 20, 2019 270 children were enrolled. All children were randomly assigned to receive placebo (n = 136) or amoxicillin (n = 134). By 14^t^ ^h^day post-operatively, total of 8 children were lost to follow-up in each arm. No major postoperative haemorrhage was registered. By 14^th^ day post-operatively, 22 (17.5%) children in the amoxicillin arm and 19 (14.8%) children in the placebo arm had reported minor haemorrhage (risk difference (RD) -2.6% (95%CI -10.2 - 5.0); p_non-inferiority_ = 0.045). By 7^th^ day post operatively, 8 (6.3%) children in amoxicillin arm and 4 (3.1%) children in placebo arm reported fever during the first week (RD -3.2% (95%CI -7.6 - 1.2); p_non-inferiority_ = 0.001). By 7^th^ day post operatively, mean pain score (mean (SD)) was 3.25 (1.53) in the amoxicillin arm and 3.56 (1.68) in the placebo arm (mean difference 0.31, (95% CI -0.02 - 0.65); p_non-inferiority_ < 0.001). No statistically significant differences between the two groups were found in any of the secondary outcomes. Findings shows, placebo is non-inferior to amoxicillin for post-operative morbidities in Tanzanian children undergoing (A)TE.

**Conclusion:**

it is recommended that antibiotics should only be used when clinically necessary to treat a specific infection. Unnecessary use of antibiotics contributes to the development of antimicrobial resistance. Trial Registration: Pan African Clinical Trials Registry PACTR201905466349317. Retrospectively registered on 15 May 2019.

## Introduction

Antimicrobial resistance (AMR) has been on a rise as a result of over prescription and misuse of antibiotics in resource limited settings [1]. Due to limited stewardship and lack of strict guidelines for antibiotic prescription, AMR as a public health threat has to be addressed locally based on scientific evidence [2,3]. AMR not only imposes high costs of obtaining superior antibiotics to treat basic infections but also endangers lives of millions of people living in these settings [4]. Children are the most vulnerable population when it comes to antibiotics misuse and over prescription due to a number of reasons, especially in limited resource settings. Diarrhoea and respiratory infections are very common in this population and are often wrongly treated with antibiotics, regardless the etiology [5]. In addition, antimicrobial surgical prophylaxis is often given in resource limited settings even if there is no evidence of its effectiveness [4-8] Amoxicillin, amongst many antibiotics, has been one of the mostly over prescribed and overused essential drugs in Africa [9]. This results into cross resistance and increasing cost of acquiring superior antibiotics, at individual and population levels. Hence, antibiotic overuse drives the development and transmission of antimicrobial resistance [2, 6, 8, 10, 11]. Prophylactic surgical use of antibiotics has been well explained in a number of guidelines in different disciplines and practices. For (adeno)tonsillectomy ( (A)TE) however antibiotics have not been advocated in a recent Cochrane review and in other reviews. Post (A)TE) antibiotics have been reserved for special situations [12-18]. However, in other settings antibiotic prophylaxis is still used post (A)TE, this is based on local practices [19-23]. In Tanzania, no available evidence supports the need of prophylactic antibiotics following (A)TE despite amoxicillin being prescribed routinely following these surgeries. As (A)TE is a major childhood surgery globally as well as in Tanzania and post (A)TE morbidities may be fatal, care should be taken on addressing them [24-28]. At the same time these life threating complications are rare and most complications are less severe and the body can overcome them without permanent sequelae. On the other hand, prophylactic antibiotics may have side effects too, some of them being severe (e.g. anaphylactic shock). The present study compares the effect of placebo versus prophylactic amoxicillin on post-operative morbidity after (A)TE in Tanzanian children.

## Methods

Study design and participants: between January 13, 2019 and September 20, 2019, we recruited eligible children aged between 2 and 14 years electively scheduled for (A)TE to participate in a two-centre, double-blinded randomized placebo-controlled, non-inferiority trial. Indications being either recurrent tonsillitis as defined as 5 or more episodes of tonsillitis per year for at least 2 consecutive years and/or obstructive sleep apnoea due to adenotonsillar hypertrophy not responding to pharmacotherapy. We excluded all children scheduled for (A)TE due to other indications or with specific co-morbidities as mentioned in our protocol [29]. The study was performed at two referral centres in northern Tanzania. Ethical approval secured from each participating hospital´s ethical committee and National Institute of Medical Research. The trial was registered in the Pan African Clinical Trials Registry (identifier: PACTR201905466349317) and the trial protocol was previously published [29]. This trial was reported according to CONSORT 2010 guidelines [30].

**Randomisation and masking**: participants received either amoxicillin 50mg/kg body weight or placebo every eight hours for 5 days after surgery. A web-based randomization module was used in a 1: 1 ratio and patients were randomized after obtaining written informed consent. Stratified randomization was accomplished using gender, age group (2-4 years, 5-8 years and 9-14 years), residence (rural or urban) and research centers as strata. All patients and health care professionals involved in the study, i.e. all other doctors at the ENT department of both hospitals as well as all nurses and microbiology staff involved were blinded to the randomization outcome. Only the pharmacist (HS) who had to provide the amoxicillin or placebo and the study supervisor, who was not involved in actual care of patients (NvH) had access to the randomization module.

**Procedures**: placebo was prepared under controlled environment by the study pharmacist. Capsules of amoxicillin were opened and active ingredients were replaced with the same amount of glucose powder. Similarly, for syrup amoxicillin, the syrup was replaced with same amount of sugar syrup maintaining bottle type, labelling and seal. All (A)TE were carried out under general anaesthesia with the use of orotracheal intubation. Procedures were performed by either residents or consultants. Technique used was based on the surgeon´s experience and preference, i.e. routine dissection, guillotine technique or electrodissection with a standardized 20-Watts monopolar technique. Both study arms were prescribed oral acetaminophen and ibuprofen post operatively. Children were kept for observation for 24-48 hours before discharged home. Following surgery, parents/caretakers were given a home-based questionnaire to be completed daily for 7 days. Body temperature and Wong-Baker? visual analogue pain scores were monitored by parents/caretakers every eight hours. Thermometers were provided. Haemorrhage, time until normal diet was resumed, time until normal activities were resumed and adverse events, such as a rash, vomiting, diarrhea and anaphylaxis were registered in the questionnaire. Children were scheduled for 2 outpatient visits at day 7 and 14 postoperatively. During each visit detailed information on progress were captured in a structured case report form. Complications, if any, were registered. A study dedicated mobile phone number was accessible to all study participants for 24 hours a day during the whole study period. Surface tonsillar swabs were obtained intraoperatively and on day 14 postoperative respectively for microbiology using Copan® Liquid Amies Elution Swab (ESwab, COPAN, Brescia, Italy). All swabs were immediately submitted to the microbiology research lab and processed according to the current international microbiology guidelines.

**Outcomes**: primary outcomes were postoperative haemorrhage, both major (defined as necessitating blood transfusion and/or return to theatre) as well as minor (defined as spitting of blood or blood stained saliva postoperative) during the first 14 days following surgery, and postoperative fever (defined as temperature greater than 38°C on 2 consecutive post-operative days or greater than 39°C on any postoperative day during the first 7 days following surgery) and the mean Wong-Baker® visual analogue pain score during the first 7 days following surgery. Secondary outcomes included; number of days until normal diet was resumed, daily activities at the 5^th^ day following surgery as described by Linden *et al*. [31] and microbial recolonization of the tonsillar beds at 14 days post A (TE).

**Statistical analysis**: the primary objective was non-inferiority of placebo compared with amoxicillin for postoperative haemorrhage (at 14 days), postoperative fever (at 7 days), and pain (at 7 days). The non-inferiority margins were based on clinically meaningful changes. The non-inferiority margins for postoperative haemorrhage, postoperative fever, and pain, were set at 5%, 5%, and 1 point on the Wong-Baker? visual analogue pain scale, respectively. Sample size calculations were performed for each of the primary outcomes, the largest number of participants needed was used. Based on the latest Cochrane Review, 2.5% of all patients who undergo an elective tonsillectomy have a significant postoperative haemorrhage [32]. To show non-inferiority with 80% power, we calculated that 121 patients per treatment arm (242 in total) were required, assuming no difference in outcomes between placebo and amoxicillin, a 5% margin of non-inferiority, and 0.05 one-sided significance level. Assuming 10% dropout, we estimated that 270 randomly assigned patients per treatment group would be needed. Continuous variables were presented as means and standard deviation or median and interquartile range (IQR) if not normally distributed. Categorical data were presented as a number with percentage. Differences between the observed risks of events (for primary outcomes of bleeding and fever) between the placebo and antibiotics groups were calculated along with one-sided 95% confidence intervals. Absolute risks are also presented as it is important to be aware of the underlying risk of events. Differences in pain score between the two treatment arms were calculated as mean differences with one-sided 95% CIs, using Student´s t-tests. Secondary outcomes were analysed using chi-squared tests for categorical data (return to normal activities and microbial recolonization) or Wilcoxon rank-sum tests (days to return to normal diet) for continuous data. P-values < 0.05 were considered statistically significant. All analyses were done according to the intention-to-treat principle. Statistical analyses were performed using R version 3.6.1 (R Foundation for Statistical Computing, Vienna, Austria).

## Results

**Figure 1:**
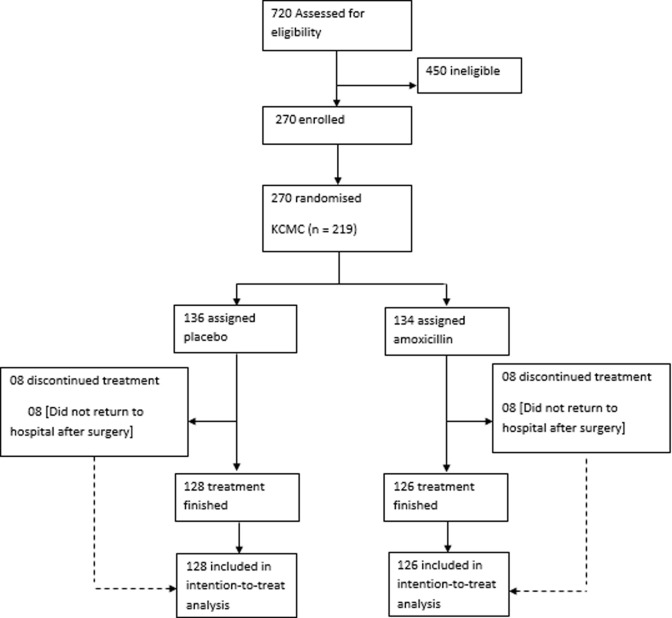
flow diagram of the trial profile

During the study period 720 (A)TEs were performed, 270 children were enrolled after meeting the inclusion criteria. Informed consent was obtained from all parents. Subsequently, 136 and 134 children were randomly assigned to receive placebo or amoxicillin, respectively. Eight children were lost to follow-up in each arm by the 14^th^ day postoperative (Figure 1). Baseline characteristics of the trial participants are shown in Table 1. In both arms, the majority of children were urban inhabitants and in preschool age. The majority of participants was treated at KCMC (81.1%). Clinically, participants scored between Brodsky tonsil grade 2 and 4. In both arms, majority of surgeries were performed by residents, with electrodissection being the preferred technique. No major postoperative haemorrhage was registered throughout the study period. Minor haemorrhage was observed in 22 (17.5%) participants in the amoxicillin arm and in 19 (14.8%) participants in the placebo arm (risk difference (RD) -2.6% (95% CI -10.2 - 5.0); p_non-inferiority_= 0.045; Table 2). During the first 7 days following surgery, 8 (6.3%) participants in the amoxicillin arm and 4 (3.1%) participants in the placebo arm had fever (RD -3.2%, (95% CI -7.6 - 1.2); p_non-inferiority_ = 0.001; Table 2). The seven days average pain score was found to be similar in both groups 3.25 (SD 1.53) and 3.56 (SD 1.68) for amoxicillin and placebo groups respectively; mean difference 0.31 (95% CI -0.02-0.65); p_non-inferiority_≤ 0.001.

**Table 1:** baseline characteristics

Characteristics	Amoxicillin (N=134)	Placebo (N=136)
Age at surgery(years) (mean (SD))	4.78 (2.25)	4.92 (2.52)
**Sex**		
Female	69 (51.5%)	58 (42.6%)
Male	65 (48.5%)	78 (57.4%)
Body Weight (kg) (mean (SD))	18.94 (6.46)	18.91 (6.31)
**Education**		
Pre-school	68(50.7%)	67(49.3%)
Primary	54(40.3%)	61(44.9%)
Not enrolled	12(9.0%)	8(5.9%)
**Residency**		
Rural	54(40.3%)	73(53.7%)
Urban	80(59.7%)	63(46.3%)
**Indication**		
Recurrent chronic tonsillitis	25 (18.7%)	25(18.4%)
OSA	46 (34.3%)	48(35.3%)
Both	63(47.0%)	63(46.3%)
**Brodsky tonsil grading score**		
Grade 2	5(3.7%)	2(1.5%)
Grade 3	62(46.3%)	62(45.6%)
Grade 4	67(50.0%)	72(52.9%)
Previous ENT surgery	2(1.5%)	4(2.9%)
**Surgical modality**		
Classical dissection	18 (13.4%)	15(11.0%)
Guillotine	4(3.0%)	7(5.1%)
Electrodissection	111(82.8%)	113(83.1%)
Combined	1(0.7%)	1(0.7%)
**Cadre surgeon**		
Resident	100(74.6%)	93(68.4%)
Registrar	27(20.1%)	25(18.4%)
Specialist	7(5.2%)	18(13.2%)
**Site**		
ALMC	25(18.7%)	26(19.1%)
KCMC	109 (81.3%)	110(80.9%)

ENT=Ear, Nose and Throat. ALMC= Arusha Lutheran Medical Centre. KCMC= Kilimanjaro Christian Medical Centre. OSA=Obstructive Sleep Apnoea.

**Table 2:** primary outcomes

	Amoxicillin (N=126)	Placebo (N=128)	Effect size (95% CI)	Pnon-inferiority
Major haemorrhage^	0(0%)	0(0%)	0 % (-1.3 - 1.3)	<0.001
Minor haemorrhage	22 (17.5%)	19 (14.8%)	-2.6% (-10.2 - 5.0)	0.045
Fever	8 (6.3%)	4(3.1%)	-3.2%(-7.6-1.2)	0.001
Mean pain score* (mean (SD))	3.25 (1.53)	3.56(1.68)	0.31(-0.02-0.65)	<0.001

^Effect size calculated using a continuity correction of 0.5; * Wong-Baker FACES® Pain Rating Score. P-values are for non-inferiority of placebo versus amoxicillin

Similarly, for secondary outcomes, no statistically significant differences were found between amoxicillin and placebo arms. On average, participants resumed diet on the second day in both arms (mean difference -0.19, 95% CI -0.68 - 0.29; p=0.43; Table 2). Five days after surgery the majority of the children had resumed normal daily activities (cried occasionally, made attempt to play and usual self together) in both arms. Main tonsillar surface and core colonizing pathogens were *Staphylococcus aureus*, and *Streptococcus* in both groups. No differences were found between both groups preoperatively. At 14 days postoperative the tonsillar fossa had been recolonised with the same bacteria as present preoperatively in 16.4% of the patients in the antibiotics group and 11.9% in the placebo group. In the majority of the other patients the tonsillar fossa was recolonised with other bacteria (Table 3). No statistical differences were found between the two groups. During the study period, none of the participants reported to have developed sepsis, possible side effects of antibiotic treatment or any other adverse events associated with study interventions in either arm.

**Table 3:** secondary outcomes

Variables	Amoxicillin (N=126)	Placebo (N=128)	Effect size (95% CI)	P
**Average number of days until normal diet was resumed**	2.85(1.99)	2.66(1.91)	-0.19(-0.68-0.29)	0.43
**Activities at 5^th^ day postoperative Cried constantly, inconsolable after surgery**	3(2.4%)	3(2.3%)		1†
**Fussy, did not want to play**	19(15.1%)	19(14.8%)		
**Cried occasionally, made attempt to play**	56(44.4%)	57(44.5%)		
**Usual self**	48(38.1%)	49(38.3%)		
Microbial recolonization*¶	19(16.4%)	14(11.9%)	-4.5% (-13.4-4.4)	0.32

† **Fisher's Exact Test** *¶ 234 participants (n =116 and n =118 for Amoxicillin and placebo respectively), intra-operative and post-operative swabs matched

## Discussion

To the best of our knowledge, the present study becomes the first largest trial to investigate the role of placebo versus post- (A)TE antibiotic in a Sub-Saharan African paediatric population. Likewise, in other resource limited settings, widespread and inappropriate use of antibiotics is not uncommon in Tanzania. Despite all other reasons for inappropriate antimicrobial prescription in our setting, surgical prophylaxis is projected as one among major contributors to AMR [4,7,33-35] In the present study, placebo was found to be non-inferior to antibiotics. Our findings on the effect of postoperative amoxicillin on post (A)TE morbidity are in accordance with those recently reported by Cochrane and other systematic reviews [17,32,36]. Some studies have reported even more haemorrhage in antibiotics arm. As might be perceived as early signs of infection, both post-operative fever and pain scores did not differ between study arms, as observed in other randomized controlled trials [15,18]. Although this study could not highlight on the possible side effects of prescribed antibiotics since none were found in our study, not giving amoxicillin has a positive protective advantage against the side effects which might be severe [19,36,37]. As with any study our trial has some limitations and shortcomings. Some of our outcomes were either self-reported or self-measured. We reckon that this cannot have resulted in missing any major haemorrhage. Due to our definition of minor haemorrhage (spitting of blood or blood-stained saliva postoperative) there may have been some over-reporting. Temperature was measured by the parents using a thermometer provided by the study. Here, some under-reporting may have occurred due to wrong technique of measuring the temperature despite proper instructions. We advocate that all these possible limitations will have been divided equally over both groups due to randomisation and therefore will not have affected study outcome. One of the major strengths of our study is the number of participants. None of the previously published studies used an equal or higher number of patients. In addition, we tried to use a wide range of primary as well as secondary outcomes. This study provides sufficient evidence that, antibiotics are neither appropriate nor necessary to prevent post (A)TE morbidity. This even accounts for a limited resource setting such as Tanzania where prophylactic antibiotics are given even more frequent because of poorer hygienic circumstances during surgery. As a global threat AMR is estimated to rise if no effective measures are taken especially in resource limited settings. Surgically, these antimicrobial agents have been widely misused, abused and prescribed without thorough clinical judgement [38-41]. Findings from this study will contribute to both local and global strategic approaches on addressing and combating antimicrobial misuse, with emphasis on surgical prophylaxis, further strengthening evidences on standardized process for mapping usage of antibiotics in children as in support of Aware, Watch, and Reserve (AWaRe) point prevalence [34,42]. Similar studies may need to be replicated in likely settings, especially for other surgeries, both within the field of ENT as well as other specialities, for which prophylactic antimicrobial agents are given without clearly justified clinical evidences. This will further enhance updated evidence for prophylactic antimicrobial roles, necessity and appropriateness which is either outdated and limitedly available [43].

## Conclusion

This study shows that placebo is non-inferior to amoxicillin for post (A)TE morbidities. Therefore, we advise against the use of prophylactic antibiotics in children undergoing (A)TE. This will hopefully contribute to reduction of AMR and more awareness of proper prophylactic antibiotic usage. Understanding the side effects, cost and availability of antibiotics and the burden of AMR that is further increased by over and misuse of postoperative antibiotics, their prescription during (A)TE is discouraged even in resources limited settings.

### What is known about this topic

- A Cochrane review found no role of antibiotics in preventing post (adeno)tonsillectomy morbidity; this did not include African patients, however, there are a few African studies available on the topic;
- In Tanzania, as in many other settings with limited resources, antibiotics are misused and overprescribed; misuse of antibiotics contributes significantly to the development of antimicrobial resistance (AMR);
- (Adeno)tonsillectomy is the most performed surgery in the pediatric population, the use of prophylactic antibiotics is likely a major contributing factor in the development of AMR.

### What this study adds

- To our knowledge, this is the first randomized clinical trial in sub-Saharan, Eastern, Central and Southern Africa to study and share findings on the effect of placebo versus amoxicillin on post (A)TE morbidities in a pediatric population;
- Findings from this study show that placebo is non-inferior to amoxicillin in reducing post (A)TE morbidities; these findings convince otorhinolaryngologists in Tanzania and elsewhere in sub-Saharan Africa to stop prescribing antibiotics to children electively undergoing (adeno)tonsillectomy;
- Economically, the findings will help to reduce cost burden to families and hospitals for unnecessary spending on antibiotics; in addition, by reducing the future number of antibiotic prescriptions the study findings may eventually contribute to the reduction of AMR; these findings strengthen the evidence from studies performed in developed settings, and may contribute to strategies towards antimicrobial stewardship in resource challenged settings.
